# Routine mortality surveillance to identify the cause of death pattern for out-of-hospital adult (aged 12+ years) deaths in Bangladesh: introduction of automated verbal autopsy

**DOI:** 10.1186/s12889-021-10468-7

**Published:** 2021-03-12

**Authors:** Md. Toufiq Hassan Shawon, Shah Ali Akbar Ashrafi, Abul Kalam Azad, Sonja M. Firth, Hafizur Chowdhury, Robert G. Mswia, Tim Adair, Ian Riley, Carla Abouzahr, Alan D. Lopez

**Affiliations:** 1 Directorate General of Health Services, Ministry of Health and Family Welfare, Dhaka, Bangladesh; 2 Data for Health Initiative, Vital Strategies, Dhaka, Bangladesh; 3 School of Population and Global Health, University of Melbourne, Parkville, VIC Australia; 4 Vital Strategies, New York, USA; 5 Data for Health Initiative, Vital Strategies, Geneva, Switzerland

**Keywords:** Automated verbal autopsy, Mortality statistics, Community deaths, Bangladesh, Causes of death

## Abstract

**Background:**

In Bangladesh, a poorly functioning national system of registering deaths and determining their causes leaves the country without important information on which to inform health programming, particularly for the 85% of deaths that occur in the community. In 2017, an improved death registration system and automated verbal autopsy (VA) were introduced to 13 upazilas to assess the utility of VA as a routine source of policy-relevant information and to identify leading causes of deaths (COD) in rural Bangladesh.

**Methods:**

Data from 22,535 VAs, collected in 12 upazilas between October 2017 and August 2019, were assigned a COD using the SmartVA Analyze 2.0 computer algorithm. The plausibility of the VA results was assessed using a series of demographic and epidemiological checks in the Verbal Autopsy Interpretation, Performance and Evaluation Resource (VIPER) software tool.

**Results:**

Completeness of community death reporting was 65%. The vast majority (85%) of adult deaths were due to non-communicable diseases, with ischemic heart disease, stroke and chronic respiratory disease comprising about 60% alone. Leading COD were broadly consistent with Global Burden of Disease study estimates.

**Conclusions:**

Routine VA collection using automated methods is feasible, can produce plausible results and provides critical information on community COD in Bangladesh. Routine VA and VIPER have potential application to countries with weak death registration systems.

**Supplementary Information:**

The online version contains supplementary material available at 10.1186/s12889-021-10468-7.

## Background

Bangladesh, a country with a population of over 160 million people, is presently experiencing a rapid epidemiological transition. Under-five mortality has fallen dramatically in the past two decades from 97 per 1000 live births in 1998 to 30 in 2018 [1]. Additionally, like other South Asian countries, there is an increasing importance of non-communicable diseases (NCD) as a cause of mortality and ill health due to changing diets and other lifestyle factors linked to an ageing population and urbanization [2]. These trends highlight the need for a reliable and timely routine source of cause of death (COD) data in Bangladesh to provide evidence for policymakers to appropriately and more efficiently allocate resources to improve public health.

However, Bangladesh has a poorly functioning national system of registering deaths and determining their causes. In 2016, the government estimated that 85% of deaths take place in the community, where few are registered and where physicians are not available to medically certify the COD [3]. Even when a community death is registered, the family members have a legal requirement to report a COD on the application form, which is generally unreliable and hence neither coded nor processed by the civil registration system.

Periodic household surveys are undertaken to address COD knowledge gaps, mostly using verbal autopsy (VA), but all these surveys are limited to under-five and maternal deaths [4, 5] . The Bangladesh Bureau of Statistics (BBS) maintains a nationally representative sample vital registration system, but COD data collected by BBS staff are unreliable and are not sufficiently accurate to support policy [6]. The International Centre for Diarrhoeal Disease Research, Bangladesh (icddr,b) maintains a comprehensive Health and Demographic Surveillance System (HDSS) in a rural area in Bangladesh. In this setting, VA data has been demonstrated to be a feasible and valid way to collect COD data [7]. However, data from the HDSS sites cannot be generalized to other populations in Bangladesh, nor is the practice of using physicians or trained health staff to review each VA interview to diagnose a COD likely to be replicable and sustainable across the country. In consequence, reliable COD information is not available for the majority of deaths in the country.

To address this issue, the Government of Bangladesh commenced a Civil Registration and Vital Statistics (CRVS) project in 2016 to improve birth and death notification, registration, COD identification and analysis of COD data, supported by the Bloomberg Philanthropies Data for Health Initiative (D4H) and with technical support from the University of Melbourne, Vital Strategies and other partners. A key intervention introduced through this initiative was automated VA embedded within the routine CRVS system to generate population level community COD data [3]. VA is a technique for determining the COD based on interviewing a family member close to the decedent and collecting information on the signs and symptoms experienced by the deceased prior to death using a specific, structured set of questions and has been demonstrated to yield useful policy relevant information on the leading causes of death for home deaths in particular [8, 9] . It is the only available method to obtain a probable COD where there is no opportunity for a physician to assign a medically certified COD. Automated VA is the application of VA using a tablet to collect data and a computer algorithm to assign the COD based on the signs and symptoms elicited from the questionnaire [10].

Following a pilot in Kaliganj Upazila (subdistrict) within the Gazipur District in January 2017, improved death registration and automated VA has been scaled up to 13 upazilas in all 8 divisions of Bangladesh. The roll-out of automated VA in Bangladesh is a significant undertaking that has the potential to provide valuable information on community COD to policymakers that has until now not been available. In this paper, we assess the utility of automated VA as a routine source of policy-relevant information on levels and trends of causes of death in rural Bangladesh. Firstly, we assess the plausibility of the results of over 22,500 VAs that were collected in the study sites using a stepwise plausibility analysis and software tool developed by the University of Melbourne [11, 12]. We then make use of this new source of data to understand the leading causes of community deaths in 13 upazilas across Bangladesh in order to better inform policy and practice.

## Methods

### Study setting and selection of upazilas

**Fig. 1:**
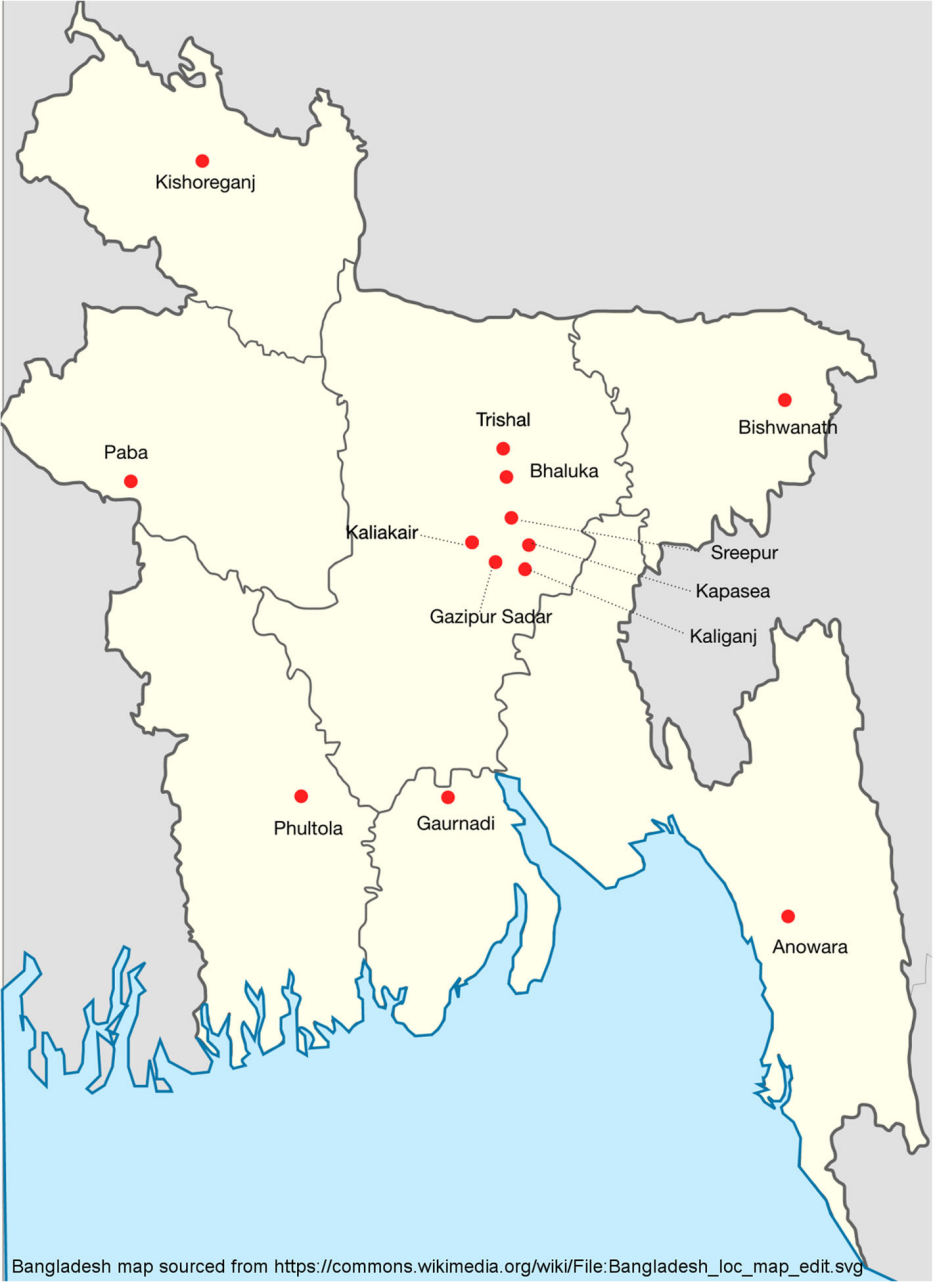
13 Upazilas in Bangladesh where verbal autopsies were carried out. Bangladesh map sourced from https://commons.wikimedia.org/wiki/File:Bangladesh_loc_map_edit.svg

The data collection was conducted in 13 upazilas in 8 divisions (see Fig. 1). These 13 upazilas, were selected purposefully and included all five upazilas of Gazipur district and at least one upazila from each of the eight divisions of the country. These upazilas are predominantly located in rural areas (63% of the population are rural residents) [3]. Most of the selected upazilas have very few private medical practitioners, and the public healthcare system, such as upazila hospital and community clinics, are the predominant healthcare delivery facilities.

We estimated the 2017 population of each upazila by projecting the 2011 Census population forward using the population growth rate between the 2001 and 2011 Censuses [13, 14] (See Additional file 1). One upazila (Gazipur Sadar) was excluded from the population projection and analysis due to an implausibly high intercensal growth (See Additional file 2). The total projected population (excluding Gazipur Sadar) was 7,213,430 or around 3% of the total Bangladesh population.

### Death identification and VA data collection

**Fig. 2:**
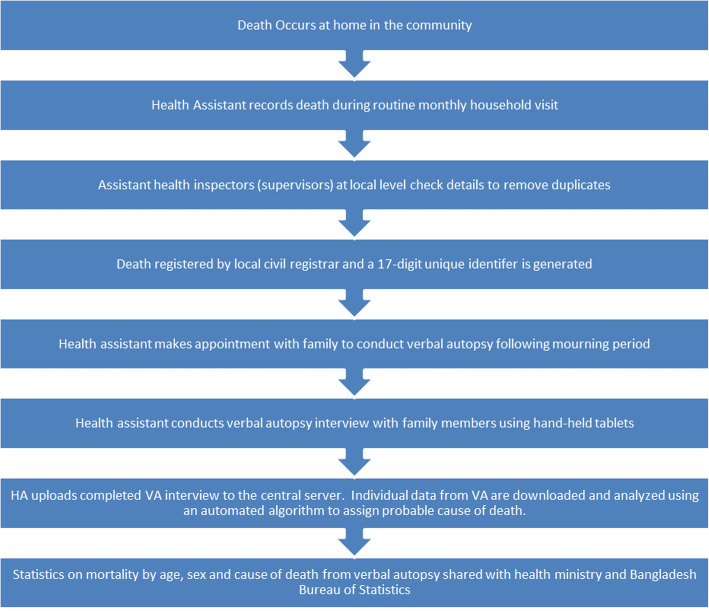
Death notification and verbal autopsy data collection process in Bangladesh

The death registration and VA administration process is described in Fig. 2.

Health Assistants (HA) with a non-medical background were orientated and tasked to identify deaths in the community during their routine household visits and to help families complete the death registration application forms and collect the associated certificates. HA are existing health staff with roles ranging from identifying antenatal care, immunization and tuberculosis needs, to health education, vitamin supplementation and helminth tablet distribution. HA are also tasked with birth and death reporting and registration. Once their supervisors had checked the data, these staff then notified the event of a death to the local registration office for official registration, preferably within 45 days of occurrence.

On receipt of the notification application, the local registrar assessed and verified the forms and entered the data into an online database, which automatically assigns a unique 17-digit death registration number to be used for issuing a death certificate. This unique number was used as the VA identification number (ID).

Once the VA death registration ID was available, the HAs arranged an appointment to meet with the family to conduct a VA interview, allowing for an appropriate mourning period, usually between one and 3 months but within 1 year of occurrence of the death. The HA were trained how to select the best respondent (usually a family member that had been involved in the care of the deceased and was there at the time of death) and conduct the VA interview using a mobile tablet. Completed interview forms were then sent to the Open Data Kit (ODK) Aggregate server using the mobile network.

### Death sample

The study included only deaths that occurred out of facility, where physicians were unavailable to certify the COD. The data collection was undertaken from October 2017 to August 2019. All the deaths in the study were registered with the local civil registrar and a death registration certificate was the pre-requisite to conduct the VA.

### VA interview data quality

Training of trainers was conducted with master trainers (doctors, nurses, medical assistants and statisticians) who subsequently trained the VA interviewers and the supervisors (existing supervisors of the HA) in each upazila. A 5 day VA curriculum was delivered covering all areas of death registration and VA including the importance of death registration, the uses of COD information, comprehension of the questions within the different modules in the questionnaire, using the tablet and ODK to collect information using the SmartVA questionnaire, how to deal sensitively with the family of the deceased, how to choose the best respondent, ethics and practicalities of collecting VA data. On the last day of the training VA Interviewers had field practice and conducted a VA, in the presence of a Master trainer. 600 HA and 52 supervisors were trained on VA. The supervisors observed VA interviews for every fifth case using a supervision checklist and checked the tablet after the interview for quality assurance. In addition, debriefing sessions with the field VA team were organized at the Upazila Health Complex to discuss their experiences and challenges, with comments and feedback provided to ensure standardized implementation of the VA interview.

### VA instrument, data management and assigning cause of death

VA data collection in this study was conducted using the Bengali version of the Population Health Metrics Research Consortium (PHMRC) shortened VA instrument [15]. This VA instrument and automated methods (Tariff method) to assign COD (together referred to as SmartVA) have been validated using a ‘gold standard’ dataset of hospital deaths on which VA was also performed [9, 16–18].

ODK version v1.10.2-v1.23.3 [19], an open-source data management software, was used to collect and store the VA data .

CODs were assigned by the Smart VA Analyze application 2.0.0 [20] which uses the Tariff 2.0 diagnostic method. The Tariff method, developed by the PHMRC, was based on the principle that certain symptoms had a stronger association with specific causes than others and that ‘tariff’ scores for each symptom-cause pair should provide sufficient information to discriminate among various potential causes of death, according to the responses from the VA questionnaire [21, 22]. The method has recently been applied in several countries implementing routine roll-out of VA to understand causes of community death [10]. The Tariff 2.0 method assigns the most probable COD from a target cause list of 33 causes of adult death, 22 causes of child death and six causes of neonatal death. Tariff 2.0 produces two main outputs; an individual COD prediction file and an aggregated VA population cause specific mortality fraction (CSMF) file. In the individual prediction file, deaths that did not have sufficient information for Smart VA Analyze to reliably predict a COD are assigned an “undetermined” COD. At the population level (represented by the CSMF), undetermined CODs are reallocated among all other specified VA CODs. Methods are described elsewhere [22].

Prior to the SmartVA analysis, the VA raw data were cleaned using an automated method that checked the VA records and removed any of the following records

1. Interview less than 1 min
2. Duplicate VA IDs
3. Mismatch in Divisions and District Codes
4. VA IDs which are not 17 Digits
5. VAs where the question ‘sex of deceased’ was refused or don’t know

### Analysis plan/VA COD plausibility analysis

To assess the plausibility of the VA data, we imported the VA analyzed data into the *Verbal Autopsy Interpretation, Performance and Evaluation Resource* (VIPER) tool [12]. This tool, and associated guidelines [11], follow four steps to assist with interpreting VA data which investigate the i) characteristics of the VA population, ii) the completeness of the VA death reporting using the empirical completeness method [23] (see Additional file 3), iii) the plausibility of the age-sex distribution of death from VA and; iv) the plausibility of the cause specific mortality fractions (CSMF) from VA. See Additional file 4 for more details on the additional inputs for VIPER.

Completeness of VA death reporting was calculated using the empirical completeness method, which uses data inputs of the number of VAs, the total VA population, the percentage of the population aged 65 years and above, and the under-five mortality rate (see further detail in Additional file 3) [23]. These data inputs reflect the key demographic drivers of the level of the crude death rate. The empirical completeness method calculates completeness of death reporting as a percentage of all deaths, i.e. both community and hospital deaths. We converted this figure to completeness as a percentage of community deaths, based on 85% of all deaths occurring in the community. Completeness (as a percentage of community deaths) was therefore calculated as completeness (as a percentage of all deaths) divided by 0.85.

### Ethical considerations

Informed verbal consent was obtained from the relatives of the deceased person, and the Ministry of Health and Family Welfare approved this consent procedure for the VA interview in Bangladesh. Participation was voluntary, and the participants had the right to refuse or withdraw at any time during data collection. Only four participants refused the interview which was always conducted in a private setting. The entire data set was kept securely and confidentially. Emotional support was offered to informants, as required. Appropriate care was taken to ensure that participants were not put at any risk; none received compensation for taking part in this study.

## Results

From October 2017 to August 2019, out of 22,535 verbal autopsies, 21,488 cases were adult (95%), 674 cases were child (3%) and 373 cases were neonate (2%). As the fraction of child and neonatal deaths were very low (compared to 11% in the comparator Global Burden of Disease [GBD] dataset), and results are likely to be biased due to low completeness in these age-groups, we have limited the COD analyses to adult deaths.

Of the 21,488 adult (12 years and above) verbal autopsies, 13,238 (61.6%) were males, and 8250 (38.4%) were females. 2384 records were discarded according to the data cleaning protocol. Over 1500 of these records were discarded due to duplicate ID (see Additional file 5).

### Population age structure

**Fig. 3:**
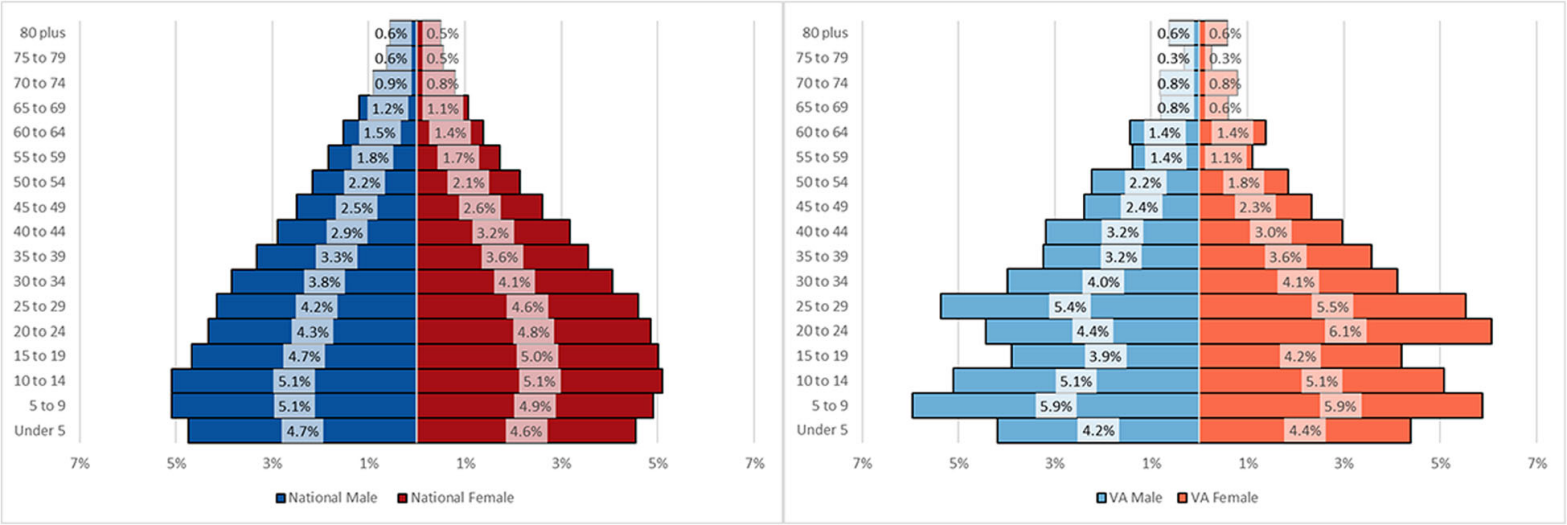
Comparison of the Bangladesh national population age-sex structure with the verbal autopsy population structure

In order to compare CSMFs from the VA population with the national estimates, it is important first to understand whether the population age structures differ in important ways that could affect COD structure. Figure 3 suggests that the age structure of the VA population is broadly similar to the national pattern, with some variation in the VA population at ages 5–9, 15–19 years and 25–29 years. However, death rates at these ages are comparatively low and hence this aberration, likely due to stochastic variation, will have little impact on overall COD patterns. The VA study population is slightly younger, with 4.8% of the population aged 65+, versus 6.2% for Bangladesh.

### Completeness of death reporting

Understanding the completeness of mortality coverage in the VA population is important since missing deaths (generally from remote or lower socioeconomic status populations) are likely to have a different cause structure to registered deaths. Across all study sites, the estimated completeness of VA death reporting for community (home) deaths was 65%, being higher in males (70%) than females (63%).

### Age-sex distribution of deaths

**Fig. 4:**
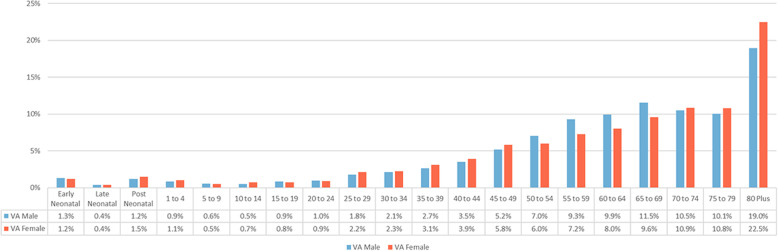
Age-sex distribution of verbal autopsy death (12 sites)

The age-sex distribution of the VA deaths is shown in Fig. 4. For adults, the main difference in age distribution between the sexes starts from age 50, with more male deaths from age 50 to 69 years contrasting with female deaths predominantly occurring after 70 years. This is consistent with the universal phenomenon of excess male mortality rates throughout adulthood [24]. The proportion of VA deaths at younger adult ages (25–59 years) was higher in VA than in GBD (see Additional file 6 - Age distribution of death, VA compared to GBD).

### Leading causes of death

We compared the CSMFs from the VA adult population with the GBD estimates for Bangladesh to assess the plausibility of overall mortality patterns suggested by VA (See Additional file 7 - Broad causes of death breakdown for adults VA compared to GBD). The broad COD structure from the GBD^1^ suggested by both sources was similar with the majority of deaths caused by NCDs (GBD Group II) - 86% and 83% for VA and GBD respectively, whilst for VA Group I accounted for a smaller fraction (6%) of all deaths compared to the GBD (11%). Injuries (GBD Group III) accounted for a similar proportion of deaths in both data sources (8% in VA;6% in the GBD).

**Fig. 5:**
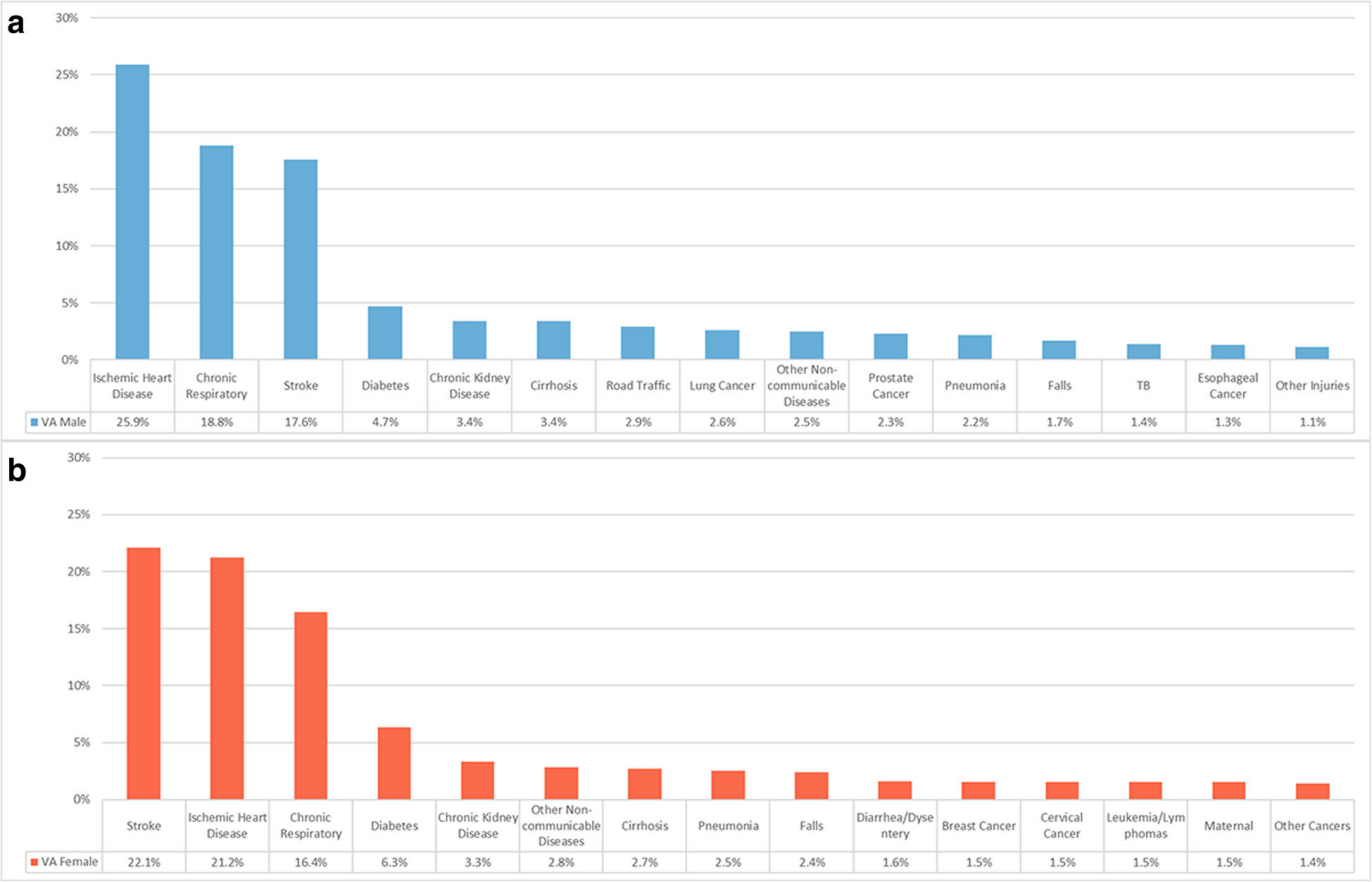
**a** Top 15 causes of death from verbal autopsy (12 sites) adult males (undetermined deaths redistributed). **b** Top 15 causes of death from verbal autopsy (12 sites) adult females (undetermined deaths redistributed)

More specifically, the top 15 causes of death in adult males according to VA (using the Tariff CSMF file) are shown in Fig. 5a. The leading cause of adult male death in Bangladesh was reported by VA to be ischemic heart disease (25.9%), accounting for about 50% more deaths than the next two causes, stroke and chronic respiratory disease. Nine of the top 10 causes are due to NCDs.

A slightly different ranking of leading causes was found for females (Fig. 5b), where stroke and ischemic heart disease each accounted for about one in five deaths, followed by chronic respiratory disease and diabetes.

Collectively, these three diseases (ischemic heart disease, stroke, chronic respiratory disease) comprise 62% and 60% of all adult deaths in males and females respectively. Diabetes, chronic kidney disease, cirrhosis and pneumonia appear in the top 15 causes for both sexes. Road traffic accident (3%), lung cancer (3%), prostate cancer (2%), esophageal cancer (1%), and TB (1%) feature in the top 15 leading causes of male death but not for women for whom falls (2%), diarrhea/dysentery (2%), maternal (2%), breast cancer (2%), and cervical cancer (2%) matter more.

**Fig. 6:**
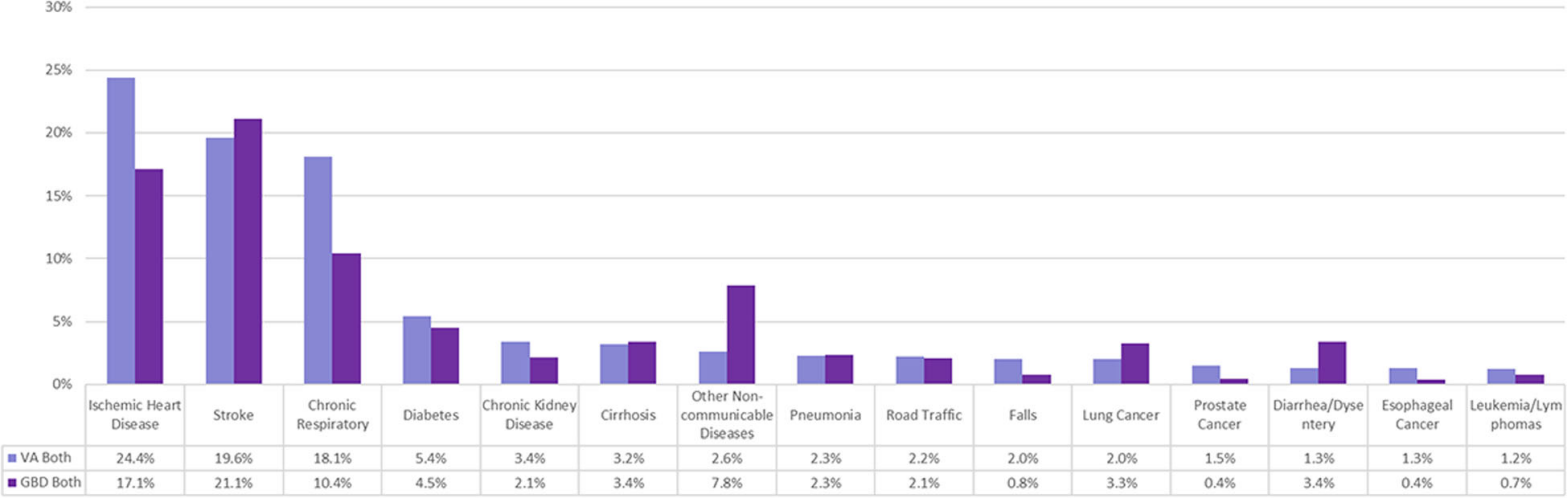
Top 15 causes of adult death from VA (12 sites) compared with GBD national estimates

The top 15 causes of death for adults suggested by VA are largely consistent with what the GBD estimates suggest for Bangladesh as a whole (Fig. 6). While there are some differences in ranking and CSMF, ischemic heart disease, stroke, chronic respiratory diseases and diabetes are the leading causes in both the VA and GBD data.

An additional plausibility check on the VA COD is presented in Table 1 which shows the age distribution of deaths for leading causes from the VA sites (using Tariff individual prediction files, ie before Undetermined COD are redistributed) compared with the GBD. Causes of death follow a highly predictable age pattern which ought to be replicated in the VA data for them to be credible. As the table confirms, deaths from most of the leading causes identified by VA follow a similar age pattern to the GBD, based on modelling decades of epidemiological data on age-patterns of causes of death [25]. The exceptions, such as observed for stroke, chronic kidney disease, diabetes, lung cancer, and prostate cancer tend to have a younger age distribution in the VA data, where deaths are less common, than at older adult ages (50–79), where the CSMFs are more similar.

**Table 1:** Age distribution of top COD in adults - VA and GBD both sexes (%) (Tariff individual predictions)

	VA					GBD				
Causes (Row Percentage)	12–49 yrs	50–59 yrs	60–69 yrs	70–79 yrs	80+ yrs	12–49 yrs	50–59 yrs	60–69 yrs	70–79 yrs	80+ yrs
Ischemic Heart Disease	20	20	24	21	15	11	17	26	25	21
Stroke	14	16	23	24	23	7	12	23	29	29
Chronic Respiratory	6	11	19	30	34	6	11	25	32	26
Diabetes	15	21	24	20	22	7	10	19	23	41
Chronic Kidney Disease	32	20	20	16	12	18	16	21	19	26
Cirrhosis	38	19	21	14	8	30	22	19	16	13
Other Non-communicable Diseases	33	16	20	12	19	17	11	13	19	40
Pneumonia	12	10	20	28	30	12	9	16	25	38
Road Traffic	60	16	9	9	6	57	16	14	8	5
Falls	16	11	20	22	31	19	8	14	24	35
Lung Cancer	17	28	33	15	7	10	18	29	27	16
Prostate Cancer	14	17	27	29	13	2	5	16	33	44
Diarrhea/Dysentery	17	8	10	27	38	10	6	14	26	44

Row percentages within each category add to 100%

The number of Undetermined deaths (deaths for which there was not enough information for Smart VA Analyze to assign an individual cause) showed an age distribution skewed towards the older age-groups (See Additional file 8 - Age distribution of Undetermined Causes of Death from VA). Overall Undetermined levels were 14% (total), 13% (males) and 17% (females).

## Discussion

To our knowledge, this is the first study to apply automated VA methods (SmartVA) to collect and diagnose information on community deaths in Bangladesh through a routine mortality system. Our findings suggest that the epidemiological transition is much more advanced in Bangladesh than might be expected given the development status of the country; approximately 85% of home deaths are caused by a NCDs, principally ischemic heart disease, stroke, chronic respiratory diseases and diabetes. We have applied a series of demographic and epidemiological checks to the VA data which suggest that SmartVA can produce plausible COD results which are consistent with established estimation efforts such as the GBD and yield valuable evidence for policy. Our findings have implications for the future use of VA in Bangladesh as an integral component of the national CRVS system designed to routinely generate detailed, reliable and timely information on community deaths.

The assessment of plausibility of the VA results was primarily based on a comparison to the GBD estimates. While the GBD cannot be considered as a ‘gold standard’, it nonetheless represents a systematic scientific effort modelling available country and regional data to provide COD estimates. They thus represent plausible descriptions of the likely age-cause pattern of mortality in populations based on observed covariates and knowledge about their established relationships with specific causes of death (e.g. smoking and lung cancer) [25]. The consistency of rankings of the top COD assigned through VA with the GBD estimates thus provides some confidence in the plausibility of the results. We would expect some differences in cause pattern between GBD and the VA datasets to reflect that GBD data includes hospital deaths. This is illustrated in the higher proportion of deaths in the GBD dataset occurring in Group I (communicable, maternal, neonatal and nutritional causes) which are more common in hospital deaths, and in a slightly lower proportion of Group III (Injury) deaths, which generally occur outside hospitals. However, the large proportion of deaths that happen in the community in Bangladesh mean that any national level data (represented by GBD) will necessarily be skewed towards community cause of death patterns. Further, the top three leading causes (stroke, ischemic heart disease, chronic respiratory diseases) are consistent with local evidence on causes of death assigned by medical assistants based on VA interviews in Matlab [7].

While the completeness of community death reporting (65%) was a marked improvement on previous practice as measured at baseline (13.5%), it was less impressive than the completeness levels obtained in the pilot study district of Kaliganj (94%) [26]. In part this may reflect the fact that the additional upazilas started at different time periods, and hence the full benefits of the VA intervention on improving registration of deaths may not have been realized. In addition, at the beginning of the study, some incentives to cover travel costs for conducting VA (usually numbering around 3–5 per month) were provided to HA. These incentives were withdrawn once the activity became a routine part of their job and may have resulted in a slight drop off in the collection of VA data. The 35% of community deaths that have not been captured by VA are disproportionately in neonatal, child and older females. Neonatal and child death registration into the CRVS is very low in Bangladesh and there is also a wide gender differential in adult deaths. Lack of knowledge or incentives to register child and neonatal deaths and the low proportion of women owning property (inheritance being a primary driver of death registration) may explain some of these disparities (Personal correspondence: Haider et al).^2^ Since registration is a pre-requisite for conducting a VA, overcoming challenges to the registration of these populations (neonatal, child and older adult females) requires further research and concerted action.

The VA CSMF results for males compared to females appear broadly plausible. Road traffic accident deaths and lung cancer, which are in the top 15 causes of male deaths, but not females, are closely linked to behaviours such as smoking which are much more prevalent among men [27, 28]. Nonetheless, while the ranking of leading causes is similar between VA and the GBD, there are some important differences, possibly due to misclassification of diseases with similar symptoms. In particular, chronic respiratory disease, which is 7.7 percentage points higher in VA than in the GBD, may be partially due to misclassification with lung cancer. Both causes share a common risk factor, namely tobacco consumption. Importantly, when cardiovascular diseases are aggregated in both datasets, they suggest an almost identical proportion of deaths; 44% in VA and 42% in GBD data.

The age distribution of the VA data does not always comply with expected patterns (represented by the GBD data), with a younger age distribution for several NCDs including stroke, chronic kidney disease, diabetes, lung cancer and prostate cancer. There is a suggestion that NCD risk factors are increasingly prevalent in younger (economically productive) years [2]. The result may also be partly due to the more frequent deaths at younger adult ages in the VA data compared to GBD. Around 11% of adult deaths were recorded without a date of birth and/or date of death, which could have resulted in some age-misreporting, although this is unlikely to have affected the age-distribution of death significantly. These unusual results warrant further investigation.

The proportion of undetermined COD (14%) in the VA data, is not unusually high and is predominantly at older ages. The higher proportion of female (17%) versus male (13%) undetermined VA is likely due to the older age distribution of death in women. VA quality is also dependent on well trained and motivated interviewers, community acceptance of the VA interview, as well as the accuracy of the computer algorithm for assigning COD. It is possible to reduce the frequency of undetermined diagnoses by improving interviewer training and implementing other quality control measures.

### Study limitation

Our study has a number of limitations that should be borne in mind when interpreting the results of the VA data. Firstly, the low number of child and neonatal deaths captured by the VA notification procedures meant that we were not able to provide a meaningful analysis of the leading causes of death for these age-groups. Effective strategies to improve the notification of child and neonatal deaths are a priority if the information base to guide policies to improve child survival is to be strengthened and fit-for-purpose.

Second, since the BBS did not have 2017 population estimates for individual upazila, we used population projection of individual upazilas from recent censuses. For one upazila (“Gazipur Sadar”), the population projection was too high, and as a result, the completeness calculations were biased downwards. We therefore excluded the population of Gazipur Sadar from the analysis, although there is no reason to believe that the VA CSMF were affected since only a small number of VAs were collected there. A related issue is that population estimates for the study population may be slightly low due to the 2011 Census population data on which they are based being under-reported. A consequence is that the true completeness of community death reporting may be slightly lower than the 65% we report.

Finally, although our study was based on a comparatively large number of cases (21,488 adult VA), some CSMFs were based on small numbers of deaths. As such, some of the smaller CSMFs in specific age-groups are going to be subject to uncertainty due to small numbers. This does not affect the confidence we have in the overall level and ranking of the top causes in our study populations.

## Conclusion

As Bangladesh has no established system to obtain mortality data with a reliable COD, VA will be critical to obtain reliable information on the health conditions of the (largely) rural population. Our study has confirmed that automated VA methods produce results that are plausible and suggests that the epidemiological transition is quite far advanced in the country where NCDs appear to be by far the leading causes of death in adults even in rural areas of Bangladesh, with much smaller contributions from injuries and infections. Notwithstanding the potential impact of the Covid-19 pandemic on mortality patterns in Bangladesh, our results indicate an urgent need to reorientate public health systems for rural Bangladeshis to incorporate a much greater focus on the prevention and control of major vascular diseases, chronic respiratory conditions, and diabetes.

Our findings also highlight the need for any scale up efforts for automated VA to place much greater emphasis on cost-effective methods to identify and diagnose child and neonatal deaths as well as improve notification of female deaths. Despite these limitations, automated VA methods such as SmartVA are feasible in Bangladesh and should be rapidly scaled up within the CRVS system to enable routine application to determine the leading causes of community deaths in various parts of the country. This would be a critically important step in establishing sound data collection practices and generating population level COD data, which will better inform health decision making and, ultimately, improve population health.

## Additional Files

**Additional file 1.** Upazila Population (2017).

**Additional file 2.** Exclusion of Gazipur Sadar from the VA analysis.

**Additional file 3.** The Empirical Completeness Calculation.

**Additional file 4.** Additional inputs into VIPER.

**Additional file 5.** Data cleaning protocol for VA data analysis.

**Additional file 6.** Age distribution of death, Verbal Autopsy (VA) compared to Global Burden of Disease (GBD).

**Additional file 7.** Broad causes of death breakdown for adults (Verbal Autopsy – Global Burden of Disease).

**Additional file 8.** Age distribution of Undetermined Causes of Death from Verbal Autopsy.

## Data Availability

The data that support the findings of this study are available from the civil registration and vital statistics system of Bangladesh, but restrictions apply to the availability of these data, which were only accessed in this study by employees of the Bangladesh Government, and are not publicly available.

## Abbreviations

BBS: Bangladesh Bureau of Statistics
COD: Cause of death
CSMF: Cause specific mortality fraction
CRVS: Civil registration and vital statistics
D4H: Bloomberg Philanthropies Data for Health Initiative
GBD: Global Burden of Disease
HA: Health Assistants
HDSS: Health and Demographic Surveillance System
icddr,b: International Centre for Diarrhoeal Disease Research, Bangladesh
ODK: Open Data Kit
NCD: Non communicable disease
PHMRC: Population Health Metrics Research Consortium
VA: Verbal Autopsy
VIPER: Verbal Autopsy Interpretation, Performance and Evaluation Resource

## Footnotes

1 GBD Level 1 causes: Group I - Communicable, maternal, neonatal and nutritional diseases; Group II – Non-communicable diseases; Group III – Injuries. (https://ourworldindata.org/burden-of-disease#the-disease-burden-by-cause)

2 Completeness of, and barriers and enablers to, death registration in rural Matlab, Bangladesh: a cross-sectional study. Under Review.

## Declarations

We declare that the content of this manuscript has not been published or submitted for publication elsewhere.
