# Supplementary material for: Routine mortality surveillance to identify the cause of death pattern for out-of-hospital adult (aged 12+ years) deaths in Bangladesh: introduction of automated verbal autopsy
Source: BMC Public Health. 2021 Mar 12;21:491. doi: 10.1186/s12889-021-10468-7 (PMC7952220; doi:10.1186/s12889-021-10468-7)
Supplement: Supplementary file 1 — Additional file 1. Upazila Population (2017). [file 12889_2021_10468_MOESM1_ESM.pdf]

Research Article: Routine mortality surveillance to identify the cause of death pattern for out-of-hospital adult (aged 12+ years) deaths in Bangladesh: introduction of automated verbal autopsy

Additional file 1: Upazila Population (2017)

Upazila	Male	Female	Total
Kaliganj	140,595	140,985	281,580
Sadar	1,514,113	1,335,426	2,849,539
Kapasia	170,867	189,956	360,824
Sreepur	307,610	297,622	605,232
Kaliakair	352,817	346,071	698,888
Trishal	220,707	234,578	455,285
Bhaluka	263,844	264,317	528,161
Phultala	43,535	46,261	89,796
Bishwanath	124,053	129,739	253,791
Paba	175,120	176,703	351,823
Kishoreganj	131,390	136,394	267,785
Gaurnadi	93,256	101,299	194,555
Anowara	128,929	147,242	276,171
Total	3,666,837	3,546,593	7,213,430

2017 population estimated by taking the 2011 Census population and projecting forward using the population growth rate between the 2001 and 2011 Censuses.
