# Supplementary material for: Routine mortality surveillance to identify the cause of death pattern for out-of-hospital adult (aged 12+ years) deaths in Bangladesh: introduction of automated verbal autopsy
Source: BMC Public Health. 2021 Mar 12;21:491. doi: 10.1186/s12889-021-10468-7 (PMC7952220; doi:10.1186/s12889-021-10468-7)
Supplement: Supplementary file 2 — Additional file 2. Exclusion of Gazipur Sadar from the VA analysis. [file 12889_2021_10468_MOESM2_ESM.pdf]

Research Article: Routine mortality surveillance to identify the cause of death pattern for out-of-hospital adult (aged 12+ years) deaths in Bangladesh: introduction of automated verbal autopsy

Additional file 2: Exclusion of Gazipur Sadar from the VA analysis

This estimation assumes that the rate of population growth in 2001-11 will continue in 2011-17. However, in the upazila of Gazipur Sadar, this assumption is likely to be incorrect because its population growth rate in 2001-11 was 7.4% per annum, compared with 2.2% for all other upazilas, due to temporary rapid in-migration for manufacturing employment. Continuation of this level of population growth post 2011 is implausible and results in a level of population for the 13 upazilas that is likely far too high and results in substantial underestimation of the completeness of VA reporting. For this reason, we excluded the Gazipur Sadar population and its 861 VA deaths from the analysis; this is expected to provide a reliable estimation of completeness of VA reporting with only a relatively small reduction in VA deaths. The final study population was estimated to be 4,363,890 at baseline (2017), or about 3% of the total population of Bangladesh. (See Additional file 1– Upazila Population (2017))
