# Supplementary material for: Routine mortality surveillance to identify the cause of death pattern for out-of-hospital adult (aged 12+ years) deaths in Bangladesh: introduction of automated verbal autopsy
Source: BMC Public Health. 2021 Mar 12;21:491. doi: 10.1186/s12889-021-10468-7 (PMC7952220; doi:10.1186/s12889-021-10468-7)
Supplement: Supplementary file 3 — Additional file 3. The Empirical Completeness Calculation. [file 12889_2021_10468_MOESM3_ESM.pdf]

Research Article: Routine mortality surveillance to identify the cause of death pattern for out-of-hospital adult (aged 12+ years) deaths in Bangladesh: introduction of automated verbal autopsy

Additional file 3: The Empirical Completeness Calculation

The empirical completeness method estimates completeness of death reporting, as a percentage of all deaths, using inputs of the number of VAs, the total VA population, the percentage of the population aged 65 years and above, and the under-five mortality rate (1) Methods to project the total upazila population for 2017 are described in the main text. The percentage of the population aged 65 years and above was estimated in 2017 by projecting, for each upazila, the percentage of population in each five-year age group based on the annual change in these figures from the 2001 to 2011 Census. This projection resulted in the male population being older than the female population, which is unlikely given that women have a higher life expectancy than males in Bangladesh. (2) We therefore assumed that males and females in the VA population have the same percentage of the population aged 65 years and above (4.77%), as consistent with the United Nations World Population Prospects estimates for Bangladesh. (2) The under-five mortality rate for the VA population was calculated as follows:

1) For each upazila, an under-five mortality rate was calculated as the average from the 2011 (3) and 2014 (4) Demographic Health Surveys for the upazila's division,
2) A preliminary under-five mortality for the VA population was then calculated as the average of these upazila under-five mortality rates weighted by the number of VA deaths in each division.
3) The final under-five mortality rate was then calculated as the preliminary under-five mortality rate for the VA population divided by the average Bangladesh under-five mortality rate from the 2011 and 2014 Demographic and Health Surveys multiplied by the 2017 Bangladesh under-five mortality rate estimated by the Inter-agency Group for Mortality Estimation (IGME) (5).

1. Adair T, Lopez AD. Estimating the completeness of death registration: An empirical method. PloS one. 2018;13(5):e0197047-e.
2. United Nations, Department of Economic and Social Affairs,, Population Division. World Population Prospects 2019. 2019.
3. National Institute of Population Research and Training. Bangladesh Demographic and Health Survey 2011 Dhaka, Bangladesh2013 [Available from: https://dhsprogram.com/pubs/pdf/fr265/fr265.pdf.
4. National Institute of Population Research and Training. Bangladesh Demographic and Health Survey 2014 Dhaka, Bangladesh2016 [Available from: https://dhsprogram.com/pubs/pdf/FR311/FR311.pdf.
5. IGME UN Inter-agency Group for Child Mortality Estimation. Under-five Mortality Rate 2018 [Available from: https://childmortality.org/data/Bangladesh.
