# Supplementary material for: Routine mortality surveillance to identify the cause of death pattern for out-of-hospital adult (aged 12+ years) deaths in Bangladesh: introduction of automated verbal autopsy
Source: BMC Public Health. 2021 Mar 12;21:491. doi: 10.1186/s12889-021-10468-7 (PMC7952220; doi:10.1186/s12889-021-10468-7)
Supplement: Supplementary file 4 — Additional file 4. Additional inputs into VIPER. [file 12889_2021_10468_MOESM4_ESM.docx]

**Research Article: Routine mortality surveillance to identify the cause of death pattern for out-of-hospital deaths in Bangladesh: introduction of automated verbal autopsy**

Additional file 4: Additional inputs into VIPER

In addition to the verbal autopsy (VA) data with assigned COD, VIPER requires a number of population inputs for the plausibility analysis. VIPER uses population data to calculate the completeness of VA reporting and to assess whether the characteristics of the population of the VA implementation areas (“VA population”) compared to the national population may help to explain the age-sex distribution of VA deaths and their CSMFs.

To estimate the completeness of VA reporting, that is, the percentage of community deaths in the VA population for which there was a VA, we used the empirical completeness method (1). We estimated total community deaths as estimated total deaths (i.e. reported deaths divided by completeness) minus an estimate of the number of hospital deaths (assumed to be 15% of all deaths), and then calculated completeness as a percentage of these community deaths (2) See Additional file 3 for further details.

The estimate of completeness of VA death reporting needs to be based on an annualized number of VAs. However, upazilas did not start data collection on the same date. We annualized VAs by calculating the weighted average VA data collection period for the 12 upazilas (based on weighted average VA start and finish dates), and then divided the number of VAs by this VA data collection period in (years). Table A4 shows the calculation of the weighted average of VA start and finish dates for the 12 upazila (excluding Gazipul Sadar).

Table A4. Calculation of weighted average of first and last verbal autopsy

| Upazila | **Median** | **Earliest** | **Latest** | **Deaths** |
| --- | --- | --- | --- | --- |
| Kaliganj | 28-02-18 | 13-03-17 | 31-08-19 | 2,598 |
| Kaliakair | 23-07-18 | 14-09-17 | 31-08-19 | 2,505 |
| Kapasia | 17-04-18 | 12-10-17 | 27-08-19 | 3,320 |
| Sreepur | 19-03-18 | 11-10-17 | 31-08-19 | 3,277 |
| Trishal | 15-03-18 | 31-10-17 | 01-09-19 | 3,230 |
| Bhaluka | 12-05-18 | 22-11-17 | 31-08-19 | 1,162 |
| Paba | 29-11-18 | 27-12-17 | 31-08-19 | 923 |
| Kishoreganj | 27-01-19 | 31-12-17 | 31-08-19 | 1,118 |
| Bishwanath | 04-09-18 | 26-11-17 | 31-08-19 | 1,720 |
| Phultala | 20-05-18 | 30-10-17 | 29-08-19 | 1,796 |
| Anowara | 23-12-18 | 29-11-17 | 31-08-19 | 872 |
| Gaurnadi | 21-01-19 | 11-10-17 | 31-08-19 | 405 |
| **Weighted average date** | **07-06-18** | **02-10-17** | **30-08-19** |  |
| **Total deaths** |  |  |  | **22.926** |

1. Adair T, Lopez AD. Estimating the completeness of death registration: An empirical method. PloS one. 2018;13(5):e0197047-e.

2. Uddin M, Ashrafi SAA, Azad AK, Chowdhury A, Chowdhury HR, Riley ID, et al. Improving coverage of civil registration and vital statistics, Bangladesh. Bull World Health Organ. 2019;97:637-41.
