# Supplementary material for: Routine mortality surveillance to identify the cause of death pattern for out-of-hospital adult (aged 12+ years) deaths in Bangladesh: introduction of automated verbal autopsy
Source: BMC Public Health. 2021 Mar 12;21:491. doi: 10.1186/s12889-021-10468-7 (PMC7952220; doi:10.1186/s12889-021-10468-7)

Research Article: Routine mortality surveillance to identify the cause of death pattern for out-of-hospital adult (aged 12+ years) deaths in Bangladesh: introduction of automated verbal autopsy

Additional file 5: Data cleaning protocol for VA data analysis

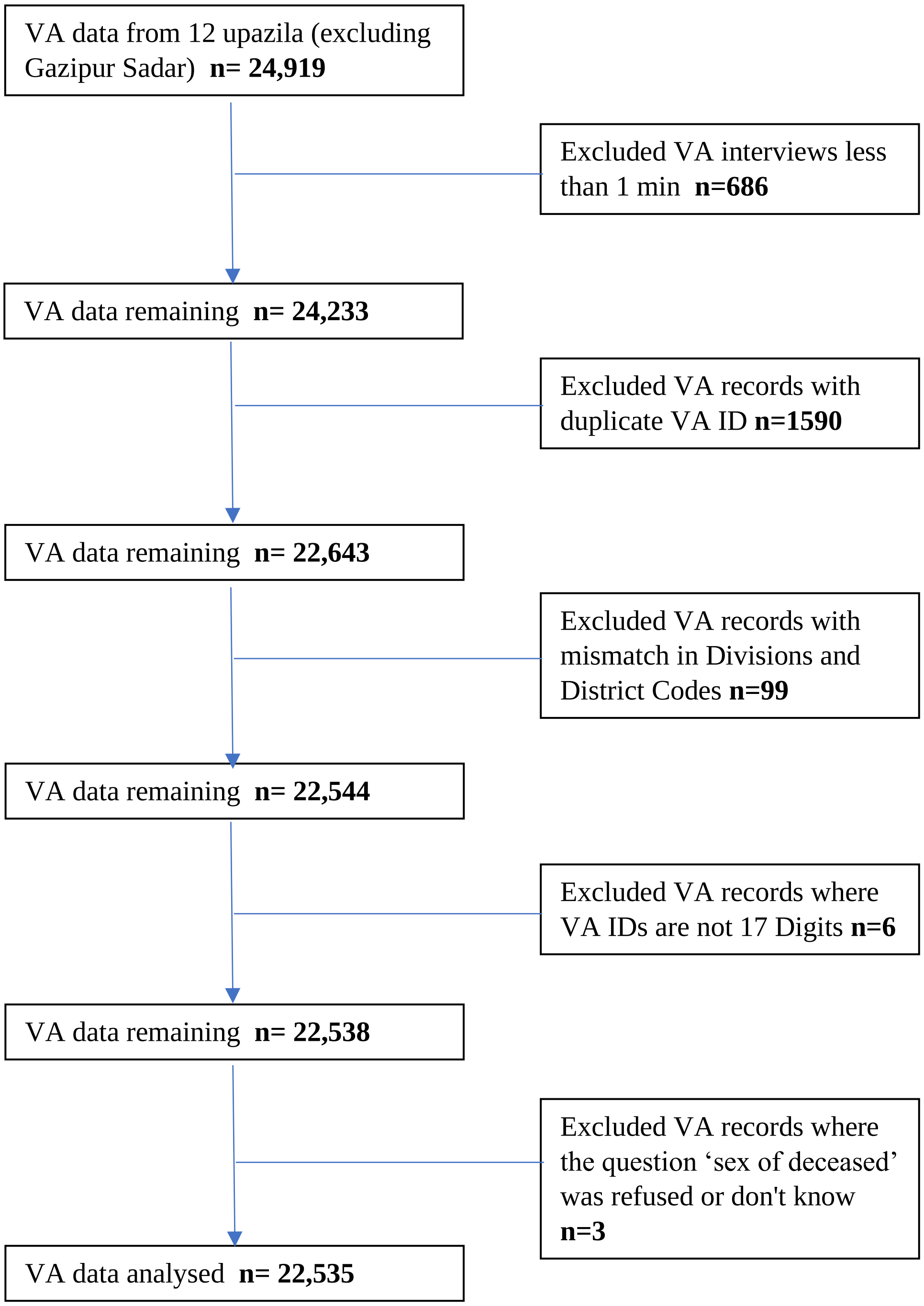

Supplement: Supplementary file 5 — Additional file 5. Data cleaning protocol for VA data analysis. [file 12889_2021_10468_MOESM5_ESM.pdf]
