# Supplementary material for: Routine mortality surveillance to identify the cause of death pattern for out-of-hospital adult (aged 12+ years) deaths in Bangladesh: introduction of automated verbal autopsy
Source: BMC Public Health. 2021 Mar 12;21:491. doi: 10.1186/s12889-021-10468-7 (PMC7952220; doi:10.1186/s12889-021-10468-7)
Supplement: Supplementary file 6 — Additional file 6. Age distribution of death, Verbal Autopsy (VA) compared to Global Burden of Disease (GBD). [file 12889_2021_10468_MOESM6_ESM.pdf]

Research Article: Routine mortality surveillance to identify the cause of death pattern for out-of-hospital adult (aged 12+ years) deaths in Bangladesh: introduction of automated verbal autopsy

Additional file 6: Age distribution of death, Verbal Autopsy (VA) compared to Global Burden of Disease (GBD)

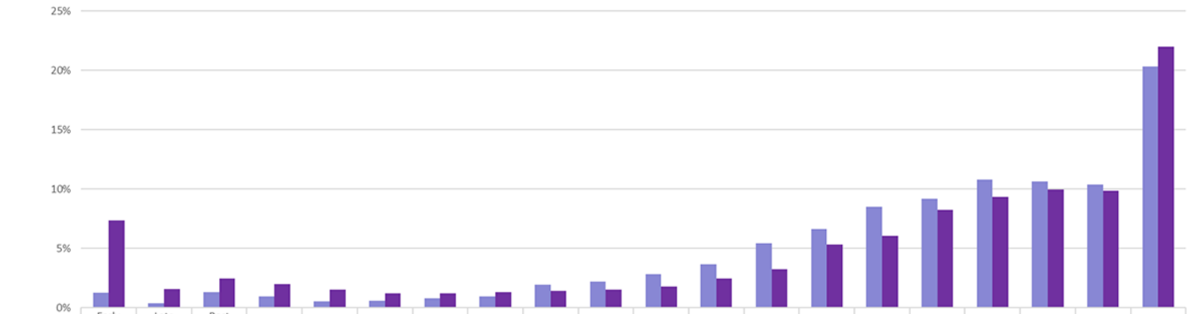

	Early Neonatal	Late Neonatal	Post Neonatal	1 to 4	5 to 9	10 to 14	15 to 19	20 to 24	25 to 29	30 to 34	35 to 39	40 to 44	45 to 49	50 to 54	55 to 59	60 to 64	65 to 69	70 to 74	75 to 79	80 Plus
VA Both	1.3%	0.4%	1.3%	0.9%	0.5%	0.6%	0.8%	1.0%	1.9%	2.2%	2.8%	3.7%	5.4%	6.6%	8.5%	9.2%	10.8%	10.7%	10.4%	20.3%
GBD Both	7.3%	1.6%	2.5%	2.0%	1.5%	1.2%	1.2%	1.3%	1.4%	1.5%	1.8%	2.5%	3.3%	5.3%	6.1%	8.3%	9.4%	10.0%	9.9%	22.0%
