# Supplementary material for: Routine mortality surveillance to identify the cause of death pattern for out-of-hospital adult (aged 12+ years) deaths in Bangladesh: introduction of automated verbal autopsy
Source: BMC Public Health. 2021 Mar 12;21:491. doi: 10.1186/s12889-021-10468-7 (PMC7952220; doi:10.1186/s12889-021-10468-7)
Supplement: Supplementary file 7 — Additional file 7. Broad causes of death breakdown for adults (Verbal Autopsy – Global Burden of Disease). [file 12889_2021_10468_MOESM7_ESM.docx]

**Research Article: Routine mortality surveillance to identify the cause of death pattern for out-of-hospital deaths in Bangladesh: introduction of automated verbal autopsy**

Additional file 7: Broad causes of death breakdown for adults (Verbal Autopsy – Global Burden of Disease)


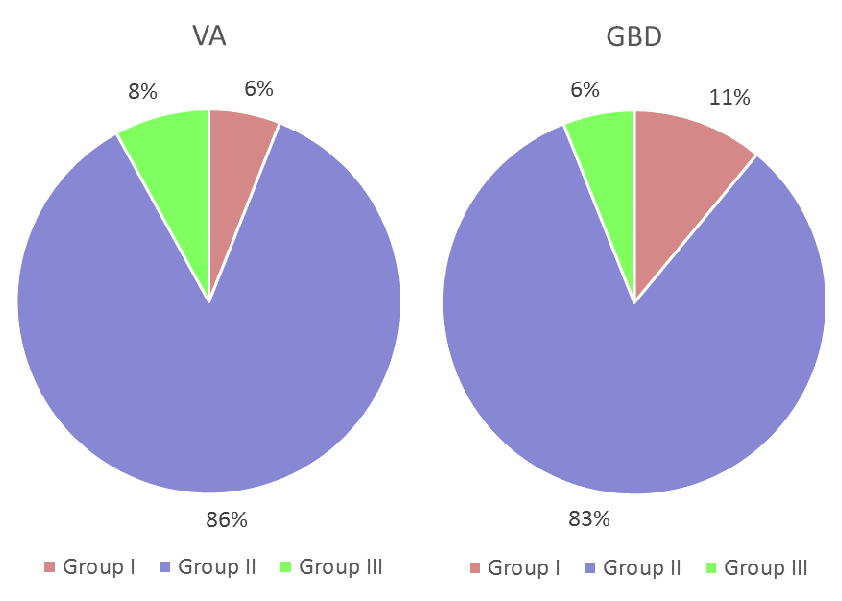


Group I - Communicable, maternal, neonatal and nutritional diseases; Group II – Non-communicable diseases; Group III – Injuries.
