# Supplementary material for: Routine mortality surveillance to identify the cause of death pattern for out-of-hospital adult (aged 12+ years) deaths in Bangladesh: introduction of automated verbal autopsy
Source: BMC Public Health. 2021 Mar 12;21:491. doi: 10.1186/s12889-021-10468-7 (PMC7952220; doi:10.1186/s12889-021-10468-7)
Supplement: Supplementary file 8 — Additional file 8. Age distribution of Undetermined Causes of Death from Verbal Autopsy. [file 12889_2021_10468_MOESM8_ESM.pdf]

Research Article: Routine mortality surveillance to identify the cause of death pattern for out-of-hospital adult (aged 12+ years) deaths in Bangladesh: introduction of automated verbal autopsy

Additional file 8: Age distribution of Undetermined Causes of Death from Verbal Autopsy

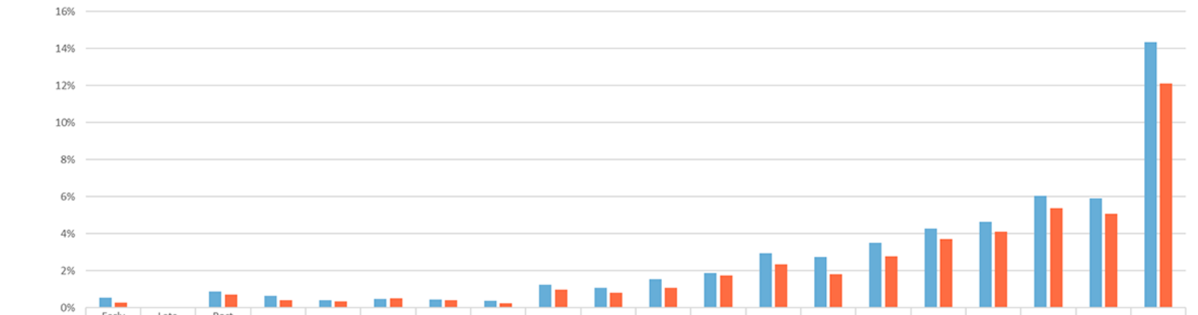

	Early Neonatal	Late Neonatal	Post Neonatal	1 to 4	5 to 9	10 to 14	15 to 19	20 to 24	25 to 29	30 to 34	35 to 39	40 to 44	45 to 49	50 to 54	55 to 59	60 to 64	65 to 69	70 to 74	75 to 79	80 plus
VA Male	0.6%	0.0%	0.9%	0.6%	0.4%	0.5%	0.4%	0.4%	1.2%	1.1%	1.5%	1.9%	2.9%	2.7%	3.5%	4.3%	4.6%	6.0%	5.9%	14.3%
VA Female	0.3%	0.0%	0.7%	0.4%	0.3%	0.5%	0.4%	0.2%	1.0%	0.8%	1.1%	1.7%	2.3%	1.8%	2.8%	3.7%	4.1%	5.4%	5.1%	12.1%
